# Supplementary material for: Polyketide synthase gene clusters in the ericoid mycorrhizal fungus Oidiodendron maius: genome-wide prediction, expression profiling, and functional characterization of knockout mutants
Source: Front Plant Sci. 2026 Sep 16;17:1924430. doi: 10.3389/fpls.2026.1924430 (PMC13623903; doi:10.3389/fpls.2026.1924430)
Supplement: Supplementary file 1 [file SupplementaryFile1.zip › Supplementary Materials/Supplementary Folder 1.zip!/Oidma1_AssemblyScaffolds_Repeatmasked/knownclusterblast/cluster16/jgi.p_Oidma1_53028_mibig_hits.html]

| MIBiG Protein | Description | MIBiG Cluster | MiBiG Product | % ID | % Coverage | BLAST Score | E-value |
| --- | --- | --- | --- | --- | --- | --- | --- |
| WP\_016325509.1 | 3-phosphoshikimate\_1-carboxyvinyltransferase | BGC0001283 | Arsenopolyketides\_biosynthetic\_gene\_cluster | 35 | 26.8987341772 | 220 | 1.1e-56 |
| AGZ15443.1 | 3-dehydroquinate\_synthase | BGC0001036 | Polyoxypeptin\_biosynthetic\_gene\_cluster | 37 | 20.1898734177 | 172 | 3.4e-42 |
| AAR98548.1 | GntB | BGC0000697 | Gentamicin\_biosynthetic\_gene\_cluster | 29 | 22.0253164557 | 143 | 2.9e-33 |
| CAF31431.1 | 2-deoxy-scyllo-inosose\_synthase | BGC0000696 | Gentamicin\_biosynthetic\_gene\_cluster | 29 | 22.0253164557 | 143 | 2.9e-33 |
| CAE06511.1 | 2-deoxy-scyllo-inosose\_synthase | BGC0000689 | 2-deoxystreptamine\_biosynthetic\_gene\_cluster | 29 | 22.0253164557 | 143 | 2.9e-33 |
| ACN38345.1 | 2-deoxy-scyllo-inosose\_synthase | BGC0000714 | Sisomicin\_biosynthetic\_gene\_cluster | 30 | 22.0886075949 | 142 | 3.8e-33 |
| ABI91458.1 | shikimate\_dehydrogenase | BGC0001094 | Enacyloxin\_biosynthetic\_gene\_cluster | 27 | 20.4430379747 | 81 | 7.9e-15 |
